# Intervention Mapping: Theory- and Evidence-Based Health Promotion Program Planning: Perspective and Examples

**DOI:** 10.3389/fpubh.2019.00209

**Published:** 2019-08-14

**Authors:** Maria E. Fernandez, Robert A. C. Ruiter, Christine M. Markham, Gerjo Kok

**Affiliations:** ^1^Center for Health Promotion and Prevention Research, University of Texas Health Science Center at Houston School of Public Health, Houston, TX, United States; ^2^Department of Work and Social Psychology, Maastricht University, Maastricht, Netherlands

**Keywords:** health promotion planning, health promotion theory, intervention mapping, implementation, planning frameworks

## Abstract

Evidence-informed health intervention planning that incorporates theoretical and empirical evidence and engages key stakeholders and community members or patients in the planning process results in interventions that are more effective. Nevertheless, exactly how and when to use evidence, theory, and community-based participation during planning represents a challenge. In this Perspective, we describe Intervention Mapping (IM), a framework for theory- and evidence-based health promotion program planning that addresses this challenge by providing a systematic and stepwise approach to planning interventions. IM has been used to develop health promotion interventions and implementation strategies in community and clinical settings globally, with over 1000 published articles employing the framework. In this Perspective, we also highlight recent and innovative applications of IM described in the articles of the Frontiers in Public Health Special Topic on IM. We conclude by discussing new directions in the application of IM including novel methods for identifying determinants of behavior and environmental conditions, the application of IM for planning implementation strategies, and IM for adaptation of evidence-based programs in new settings.

## Introduction

The development of effective health promotion interventions often requires reviews of the relevant literature, application of theories, collection of new data, and involvement of experts, community members, and stakeholders in the planning process. Applying information from these varied sources to inform intervention development presents a challenge for even well-trained health promotion practitioners. The purpose of this perspective paper is to provide an overview of Intervention Mapping, a framework for theory- and evidence-based health promotion program planning, and to highlight examples of applications of IM, as described in the articles of the *Frontiers in Public Health Special Topic on Intervention Mapping* publication (1).

IM is a planning framework that provides a systematic process and detailed protocol for effective, step-by-step decision-making for intervention development, implementation, and evaluation. It is grounded in community based participatory research methods to ensure that the intervention matches priority population needs and intervention contexts. IM takes an ecological approach to understand health problems and to intervene at multiple levels (e.g., individual, interpersonal, organization, and community) and as such guides the development of multi-level interventions.

IM provides guidelines and tools to ensure health promotion program is based on empirical evidence and sound theories. IM is also used for the planning and development of implementation strategies for program adoption, implementation, and maintenance (2). Following the IM process results in guidance and documentation of decisions at each step, charting a map from the initial steps of recognizing a need or problem through evaluation and dissemination. Compared to other protocols in health promotion planning, such as PRECEDE-PROCEED (3), Behavior Centered Design (4), and COMBI (5), IM helps planners to apply theories by linking social-cognitive determinants of behavior to methods for behavior and environmental change and by linking methods for behavior change to practical applications that operationalize these methods.

## Intervention Mapping Steps

The IM intervention development process has six steps: (1) Establish a detailed understanding of the health problem, the population at risk, the behavioral and environmental causes, and the determinants of these behavioral and environmental conditions; and, assess available resources; (2) Describe the behavioral and environmental outcomes, create objectives for changes in the determinants of behavior and environmental causes, and specify the targets of the intervention program; (3) Identify theory- and evidence-based behavior change methods that influence the determinants and translate these to practical applications that fit the intervention context; (4) Combine the intervention components into a coherent program that uses delivery channels that fit the context; (5) Develop implementation strategies to facilitate adoption, implementation, and maintenance of the program; and (6) Plan both process and outcome evaluation to assess program implementation, and efficacy or effectiveness (2).

IM thus defines and describes an iterative path from problem identification to problem solving or mitigation (2). Each of the six steps comprises several tasks, and completion of these tasks creates a foundation for the next step. Completion of all six steps results in a blueprint for designing, implementing, and evaluating the intervention.

An IM approach is characterized by three perspectives, applied during the program planning process: participatory planning, eclectic use of theory, and an ecological and systems approach for understanding health problems and intervening to address them (3). Participatory perspectives emphasize equity in decision-making and community and stakeholder engagement in all phases of planning (6, 7). From this perspective, all aspects of decision-making should involve the priority population and program implementers to ensure that the program adequately addresses community needs (6, 7). IM provides guidance at each step for how to do this. Additionally, IM guides the use of theories to understand the behavioral and environmental causes of health problems, identify their determinants, and select change methods to address them. Theories are abstractions of reality and may provide only partial explanations for understanding the causes of health problems (8, 9). In program planning, it is unlikely that one theory can sufficiently explain influences on health and provide guidance to address the causes. Thus, multiple theories are often used. IM provides a framework for incorporating multiple theories during intervention planning (10). Finally, an ecological and systems perspective recognizes that social and physical environmental conditions may have an even stronger impact on behaviors than do factors related to individuals (11).

Below, we describe IM steps and tasks and highlight key papers on IM that provide examples of how IM has been applied. Finally, we present new directions and applications for IM in the field of health promotion and beyond.

**Figure 1:**
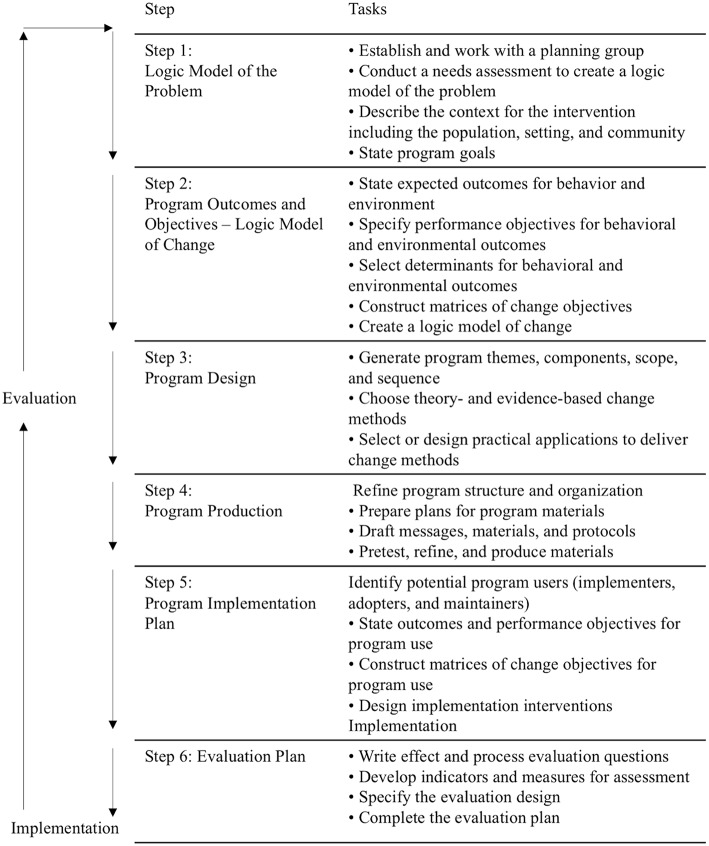
Intervention mapping steps and tasks [Bartholomew-Eldredge et al. (2), chapter 1].

**Figure 2:**
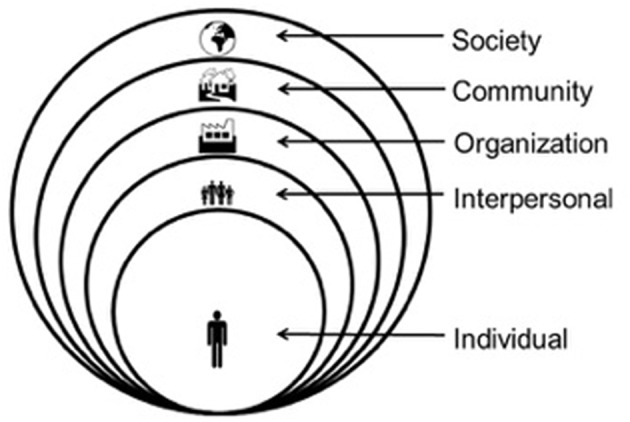
Multilevel factors influencing health.

Figure 1 shows the six steps of the IM process and their related tasks, while Figure 2 shows the environmental conditions that influence individual behavior.

### Step 1. Logic Model of the Problem

Step 1, which is based on the PRECEDE model (3), is a careful description of the problem that will enable intervention planning. The program-planning group conducts an analysis of health and quality of life, behaviors, and environmental conditions that contribute to the health problem directly or to the risk behaviors. The group also identifies factors (determinants) that influence the risk behaviors and problematic environmental conditions contributing to the health problem. This step helps planners distinguish between behaviors, environmental conditions, and their determinants, helping them better articulate and document needed changes and desired outcomes in Step 2.

### Step 2. Logic Model of Change

In Step 2, the planning group articulates the desired health promoting behaviors and environmental conditions. The group then specifies performance objectives (or sub-behaviors) for the at-risk group and for those responsible for making changes in the environment. The planning group sets performance objectives breaking down each behavior and environmental condition into subcomponents by answering certain questions: “What does the person need to do to accomplish the behavior?” and “What does the environmental agent need to do to create the environmental change?” They then identify determinants of health-promoting behaviors and environmental conditions by asking: “Why would someone do this behavior?” and “Why would someone make this environmental change?”

To make decisions about salient determinants that should be targeted with the intervention, IM guides planners through four core processes (2): (1) involving representatives from the target population, stakeholders, and implementers in brainstorming in the planning group; (2) searching through empirical literature for determinants of behavior or environmental conditions; (3) identifying and applying pertinent theories on determinants that influence these; and (4) conducting qualitative and quantitative research to explore unanswered questions. Using the information generated, the planning group sets priorities and selects a final list of determinants to target (2, 12). The group then creates a matrix of change objectives through combining performance objectives and determinants. These same core processes are use in each step of the IM process.

IM provides guidance on how to use theory to inform the development process. Planning groups can identify appropriate theories by (1) searching the literature on the health topic, (2) matching ideas from the brainstorming process to theoretical constructs, and (3) applying frequently used theories (9). The theories can guide the identification of determinants and, subsequently, the selection of methods (in Step 3) to influence these determinants. For example, while answering, “Why would someone engage in this behavior?” the planning group might brainstorm, “The person has confidence that he or she could do it,” which points to the theoretical construct of “self-efficacy” in social cognitive theory (13). This labeling of answers to the “why” question, using theory-based psychosocial constructs, leads to the selection of appropriate change methods (e.g., modeling) in Step 3.

### Step 3. Program Design

In Step 3, the planning group discusses initial ideas for the program and selects theory- and evidence-based behavior change methods based on the determinants that they need to change (2). A number of systematic reviews and meta-analyses of health promotion programs show that reasonable use of theory-based methods increases intervention effectiveness in changing behavior (14–19). In this step, program objectives are arranged or grouped by determinants. Theoretical methods that may help achieve the program objectives are identified, and then translated into practical applications or strategies. A theory-based change method is a technique for changing a behavioral determinant of an individual or environmental agent, while a practical application is a specific strategy that delivers the method in a way that fits the needs of the priority group and the program setting. Some methods can be used for several determinants, while others work only for a specific determinant (2).

There has been growing interest in systematic descriptions of health promotion interventions, their theoretical methods, and the determinants these methods are expected to change. Abraham and Michie (20), for example, created the Behavior Change Technique (BCT) taxonomy, used to identify intervention content (21, 22). Peters et al. (18) nevertheless, note that BCT taxonomies fail to describe the specific conditions or requirements that make these methods effective (23). IM, however, describes the parameters of methods that are essential for both identifying successful methods in the literature and for developing intervention components (21). For instance, modeling is effective only if reinforced and when observers pay attention, have adequate self-efficacy and skills, identify with the model, and observe a coping model instead of a mastery model (13). Each theoretical method has its own conditions for effectiveness; for example, goal setting is effective only when the selected goal is challenging but attainable (24). Fear arousal requires high self-efficacy expectations about behavior (18), which can be difficult due of the complex nature of most behavior change settings. Khan et al. (25) described processes (and their measures) that can be used by communities and local governments in planning and monitoring environmental and policy-level interventions for obesity prevention. Mesters et al. (26) notes, however, that it is often difficult to determine which components of the programs contribute to the effectiveness of the health promotion program. Moreover, inadequate reporting of theory and evidence-informed behavior change methods and their applications further limits the ability to advance the science of what works and makes program adaptation challenging. IM responds to the call for better understanding and reporting of intervention (27, 28).

### Step 4. Program Production

In Step 4, the various applications selected in Step 3 are organized and produced (2). The program planning group decides the overall structure, themes, channels, and vehicles of the program. They design and produce materials that are culturally relevant and appealing, work with other stakeholders, and pilot-test the pertinent program elements. The program planning group is responsible for correctly translating theoretical methods into practical applications, using the methods' parameters. To this end, the program planning group and production professionals (writers, video producers, graphic artists) must work together to ensure that the final program products are appealing and accessible as well as reflect the key methods, practical applications, and messages developed during the planning process. Step 4 includes pretesting and ensures the implementation of effective program materials and program fit with the particular context and population. Typically, during pretesting, comprehension, attractiveness, acceptance, believability, motivation, and preliminary indications of effectiveness are assessed, and recommendations for improvement are provided. Pretesting should be conducted after concept and message design and materials development but before materials are finalized (29, 30). It can be executed using experimental research designs (31), focus groups, in-depth interviews, and intercept surveys, among other methods.

### Step 5. Program Implementation Plan

Effective health education and promotion programs can lose their impact if they are not used before desired health impacts are achieved (32–34). IM provides a systematic process for the development of implementation strategies either for initial use of the program or for scale-up and spread of evidence-based programs already developed and tested. The use of IM to develop implementation strategies provides for the clear articulation of the mechanisms contained in these strategies, a gap in the implementation science literature (35–37). Step 5 guides the development of implementation approaches, also known as strategies or interventions. This step guides the planning group through thinking about adoption, implementation, and maintenance as well as who has to do what at each of these stages and why. Understanding the factors that influence implementation is critical for the selection of methods to address these factors.

Program implementers are the people who are responsible for the delivery of the program and can include organizational leaders responsible for program adoption and maintenance as well as those responsible for actual delivery of program materials and activities to participants. For example, nurses will present programs to patients, and teachers will deliver health education programs to students. Others in the organization or setting, even though they are not program implementers, may be responsible for making decisions about whether or not the program is adopted and for identifying individuals who will deliver the program. For example, school principals may not deliver health education curriculum; however, their support for program adoption and maintenance is critical.

IM Step 5 can be used not only to plan implementation the first time a program is developed and used but also can be used to develop plans for scale up and spread of existing evidence-based interventions. Program planning groups can address program implementers' personal determinants, like knowledge and outcome expectations for the program and self-efficacy for enacting program activities at the individual level with methods, such as persuasive communication, tailoring, and modeling. However, implementation almost always involves organizational change, which means program planning groups also have to apply methods at environmental levels. Organizational theory and implementation science frameworks can be used to understand the determinants and contextual factors that influence implementation and to guide the selection of methods that will support program implementation (38, 39).

### Step 6. Evaluation Plan

Effect and process evaluation will verify if the objectives chosen in Steps 2 and 5, respectively, have been reached, and need to be carefully planned. Previous IM steps help inform the evaluation plan since behaviors, environments, their sub-components, and determinants are clearly spelled out (2). Fernandez et al. (57) describe the use of Intervention Mapping for planning implementation strategies, a process we call *Implementation Mapping*.

## Intervention Mapping in the Real World

Special-topic authors provide examples of the application of IM across settings and topics (1). There are several examples of the use of IM for the development of eHealth interventions. Shegog and Begley (40), using IM, involved both a diverse planning group and a patient provider advisory group to develop a decision support tool (DST) to increase self-management among epilepsy patients and their care providers. The tool is used to increase awareness and efficacy of self-management behaviors among epilepsy patients and their healthcare providers and to improve communication during clinic visits. The Shegog and Begley (40) paper includes a table that illustrates the identification of methods, organized by determinants, and how these were operationalized, using practical applications of the DST. The authors demonstrate how the online decision-support system in this case can include multiple methods and practical applications to address users' determinants of self-management.

Pot et al. (41) present the application of IM in the development of a web-based, tailored intervention that promotes HPV vaccination acceptance. In their study, mothers were the target group and were systematically involved in the development process. The mothers were ambivalent about HPV vaccination, and the intervention focuses on informed decision-making. The needs, behavioral outcomes, and targeted determinants are carefully described and include examples, and the full matrices of change objectives are found in the supplementary materials. The web intervention combined freedom of choice with tunneling and virtual assistants who delivered the tailored feedback. The intervention was pilot-tested, and the implementation plan focuses on the web-based intervention owners.

Rodriguez et al. (42) and Serra et al. (43) describe the application of IM in planning interactive multimedia applications for low-income Hispanics (Mexican Americans and Puerto Ricans). Rodriguez et al. used IM in the development of an intervention for parents to increase HPV vaccination in adolescent girls. The authors also used IM steps to adapt the intervention and create a module for parents of boys. The authors select and operationalize methods targeting parents' decision-making, with implicit recognition of parameters. They also describe using IM Step 5 for the development of implementation strategies (delivery by lay health workers).

Serra et al. (43) applied IM to plan an intervention to increase colorectal cancer screening (CRCS) in Puerto Rico. The authors developed a needs and asset assessment that included a review of factors that influence CRCS among Hispanics, taking into account the preferences of the target group, and collected data. They describe objectives at the level of behavior (performance objectives) and determinants (change objectives). They identified two overarching methods: entertainment education and behavioral journalism. The intervention materials included an interactive tablet-based application, print materials, an action plan, with a follow-up phone call to determine and address remaining barriers. As in the Shegog and Begley (40) and Rodriguez et al. (42) examples, IM helped to identify determinants, and the interactive tailoring features of the intervention provided specific messages for users that depended on their beliefs, knowledge, and identified barriers. Targeting health care providers directly was not possible, but a patient activation element (patient-mediated prompts) was added to the intervention to increase provider recommendations and referrals for CRCS.

Fassier et al. (44) describe the use of IM in the development of an intervention to help breast cancer survivors in France successfully return to work after treatment. The authors emphasize the importance of taking an ecological perspective to planning and note that IM can help identify and document interpersonal, organizational, community, and societal influences. They also describe the development of the planning group, which included a broad array of stakeholders who helped to identify priorities, and environmental conditions that influenced the return to work. The paper provides an example of the use of IM in the early stages of program development to understand a problem at multiple levels, develop a logic model of change, and guide assessment.

ten Hoor et al. (45) used IM to develop an intervention to prevent obesity among Dutch adolescents. Using the socioecological approach that underlies the IM process, they identified important contributors to physical activity in the adolescent's social context, as based in social determination and social comparison theories. They also extended the theory of expanded, extended, and enhanced opportunities (TEO) for physical activity to include “enriched” opportunities, such as the incorporation of weight training into the school-based physical activity program. The paper is an example of how IM can assist in incorporating elements of different theoretical perspectives to inform program development.

Vissenberg et al. (46) used IM to develop a social network-based intervention for diabetes self-management targeted to Dutch, Surinamese, Moroccan, and Turkish families who live in the Netherlands. The authors note that underlying the challenges to self-management behavior among these populations are cultural factors and socioeconomic status. In line with IM, they recommend a greater engagement of the priority populations and other stakeholders in the planning process. The article provides an example of a logic model as derived from the IM process.

Mesters et al. (26) used IM to analyze an effective intervention to promote breastfeeding of infants at risk for asthma. The authors noted that the literature suggests certain demographic, biological, and social determinants at three time periods: prenatal, postnatal initiation, and postnatal continuation. IM was used to describe performance and to develop change objectives. Environmental factors included the mother's partner. Mesters et al. provide examples of how the process of writing performance objectives forced program planners to describe in detail the actions necessary to accomplish behaviors, which ultimately led to important content and effective strategies that may have otherwise been omitted, e.g., the inclusion of the mother's partner. An evaluation of the program showed that it resulted in positive behavioral changes, which the authors attributed to a careful analysis of the determinants and preparation for the unexpected negative attitudes of others (26).

## New Directions

Although IM provides guidance to identify needs and develop interventions, additional research and approaches are needed to more accurately address the questions posed by each of the steps including the identification of determinants and the selection of methods. Crutzen et al. (12) describe an approach for selecting determinants to target in interventions. They suggest visualization of confidence intervals and correlations. The authors clearly explain the importance of identifying determinants. They also note that currently used approaches for identifying determinants are insufficient. They propose a confidence interval-based estimation of relevance (CIBER) approach for selecting determinants to target in an intervention. In CIBER, the data are visualized as diamond plots. They presented an MDMA (ecstasy) study, in which four determinants are discussed, as an example of the use of CIBER. The statistical tool is available at no cost.

Additional research is needed to build the evidence base for the effectiveness of certain methods to address determinants. Peters et al. (18) highlight the limitations of existing taxonomies of methods derived from the meta-analyses of interventions, which include misapplied methods without consideration of parameters, confounding factors such as co-occurring methods, and the interaction of methods and context. In response, they propose an “iterative protocol for evidence base accumulation,” whereby researchers conduct meta-analyses of applied health behavior change interventions, taking into consideration the parameters of those methods. This research would then lead to basic experimental studies that test methods under various conditions. Meta-analyses of these experiments would then provide information about which methods are effective and under which conditions.

IM addresses the growing body of evidence on the influence of the environmental context on health and health behavior [e.g., (47)] by providing a robust framework for planning health interventions that considers various facets of the environment. Springer et al. (48) explore how health planners and practitioners can further incorporate the environmental context of health intervention design through the concept of *health promotion interweaving*. Building from theoretical perspectives rooted in social-ecological models, improvement science, and systems thinking, this paper advances an indigenous health intervention development approach that takes into account the environmental context, to designing interventions. While IM is specifically structured for the community and stakeholder involvement, an important contribution of this paper is its description of theory- and practice-based *interweaving* concepts (49, 50) in relation to specific environments, such as *Health in All Policies* (policy environment), *environmental print* (information environment), *appropriable organization* (social/organizational environment), and *shared use agreements* (built environment). Springer and colleagues' exploration of health promotion interweaving as a health planning approach promotes greater intentionality for designing health promotion strategies, practices, programs, and policies (47, 49, 50).

Using IM to plan implementation strategies has recently gained attention (37, 51) partly due to the challenges of applying implementation science theories and framework to inform the planning process. Highfield et al. (52) describe the use of IM to develop an implementation intervention to increase adoption, implementation, and maintenance of an existing evidence-based program to improve mammography adherence in community healthcare clinics. The goal was to scale-up the Peace of Mind Program, which had been previously adapted, using IM (53), for African American Women served by community clinics. The authors describe the steps in their process and provide examples of how implementation science theories and frameworks can inform the IM process.

Based on the recognition that evidence-based sexual health education programs are underutilized by school districts, Peskin et al. (54) used IM to create an online tool to help school districts to adopt, implement, and maintain evidence-based sexual health education programs. The authors had previously developed the Choosing and Maintaining Effective Programs for Sex Education in Schools (CHAMPS) model. They provide an example of the use IM for planning implementation interventions. This paper also provides an example of how IM can be useful in adapting evidence-based interventions so that they can be delivered through different platforms.

The use of IM as a tool in implementation science for the development of implementation strategies is growing (55–57). Using IM increases the ability to map strategies to specific barriers and facilitators of implementation, with a particular focus on the mechanisms and methods that will bring about the needed changes. Thus, the application of IM in the development of implementation strategies can address a knowledge and practice gap in implementation science.

## Conclusion

IM helps health promoters to develop well-thought-out theory- and evidence-based programs through the identification of key changeable determinants of risk behaviors, the choice of appropriate intervention methods and applications, and the development of implementation strategies to ensure use and dissemination. Although the IM process is described through its sequential steps, IM is intended to be iterative, and, indeed, most of the studies presented above describe the IM planning process this way. Throughout the process, planners gain new knowledge about the population, determinants, environment, and/or effective and appropriate methods that sometimes requires cycling through earlier steps to expand or refine the program.

The IM protocol assists program-planning groups to optimize the chances of program effectiveness, and IM has been utilized widely across multiple health domains, populations, and settings all over the world. The use of IM can make health education and health promotion programs stronger, more effective, and more widely disseminated to improve the impact of public health programs.

## Author Contributions

MF, RR, CM, and GK contributed to the manuscript conceptualization, discussion of new directions and the conclusions. GK and MF contributed to the description of studies in the special topics issue.

### Conflict of Interest Statement

The authors declare that the research was conducted in the absence of any commercial or financial relationships that could be construed as a potential conflict of interest.
